# Evidence of Magnetic Inversion in Single Ni Nanoparticles

**DOI:** 10.1038/srep36156

**Published:** 2016-11-08

**Authors:** W. Jiang, P. Gartland, D. Davidović

**Affiliations:** 1 School of Physics, Georgia Institute of Technology, Atlanta, GA 30332, USA.

## Abstract

Superparamagnetism is an unwanted property of small magnetic particles where the magnetization of the particle flips randomly in time, due to thermal noise. There has been an increased attention in the properties of superparamagnetic particles recently, because of their potential applications in high density storage and medicine. In electron transport through single nanometer scale magnetic particles, the current can also cause the magnetization to flip randomly in time, even at low temperature. Here we show experimental evidence that when the current is then reduced towards zero in the applied magnetic field, the magnetization can reliably freeze about a higher anisotropy-energy minimum, where it tends to be inverted with respect to the magnetic field direction. Specifically, we use spin-unpolarized tunneling spectroscopy of discrete levels in single Ni particles 2–4 nm in diameter at mK-temperature, and find that the the magnetic excitation energy at the onset of current decreases when the magnetic field increases, reaching near degeneracy at nonzero magnetic field. We discuss the potential for spintronic applications such as current induced magnetization switching without any spin-polarized leads.

Over the past several decades, the properties of small ferromagnetic particles have frequently been described using the macrospin approach, where the spin of the particle is uniform in space^1^,^2^,^3^,^4^ and the elementary spin excitation is the uniform magnetization mode, or the ferromagnetic resonance (FMR). In the Stoner-Wohlfarth model of magnetization reversal in the static applied magnetic field, the total classical spin of the particle 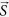 rotates while its magnitude is constant. The applied magnetic field tends to decrease the depth of the potential-well about the metastable anisotropy energy minima, and at the switching field the depth approaches zero. The energy of the FMR about metastable minima will also decrease versus magnetic field, approaching negligibly small value at the switching field. This behavior is contrasted with that about the stable magnetic minimum, where both the potential-well and the FMR energy tend to increase with the magnetic field.

In this article we measure the magnetic field dependence of the elementary magnetic excitation energy in single Ni particles, by measuring the zero-field splitting (ZFS) of discrete electron-box levels at 30 mK temperature. ZFS in the tunneling spectra of magnetic quantum dots is considered to be a signature of magnetic states with non-zero magnetic anisotropy. Sequential and inelastic electron tunneling spectroscopy in single magnetic molecules displayed ZFS with energies in the range 0.1–1 meV^5^,^6^. The splitting energy was an increasing function of the magnetic field, consistent with magnetic excitations above the stable magnetic ground state of the molecule. ZFS has also been observed in quantum dots made from transition metal ferromagnetic particles, and was attributed to the magnetic anisotropy contribution to the energies of the tunneling transitions between states with spin >1/2^7^,^8^. In this report we also find ZFS in single Ni particles. However, in two out of the five measured samples, we find that the ZFS is decreasing with the magnetic field, and degeneracy is approached at a nonzero magnetic field. As we discuss in this report, such property suggests that the spin of the Ni particle is localizing about the metastable state, as the Fermi level in the lead passes through the energy range of a tunneling transition between discrete electronic levels in the particle. We refer to this effect as magnetic inversion. We show theoretically that such inversion is possible in the macrospin model and manifested by the nonzero spin degeneracy field consistent with our measurements. The inversion is analogous to the effect of population inversion in laser physics, where optical pumping and a particular hierarchy of relaxation rates between those states, favor inverted population.

## Device Description

**Figure 1:**
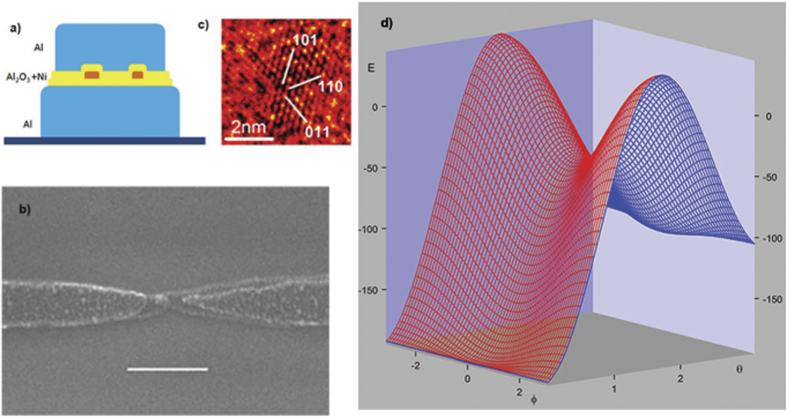
Device description: (**a)** Schematic of our tunneling junction containing nanometer scale Ni particles. (**b)** Scanning electron microscope image of a typical device. The bar indicates 200 nm. (**c)** Transmission electron microscope image of a Ni particle on the Al_2_O_3_ surface. (**d)** Classical anisotropy energy landscape for the model Hamiltonian used in the text.

The schematic of our tunneling device is shown in Fig. 1a. The device is a sandwich containing two Al leads as bottom and top layer, and an insulating Al_2_O_3_ tunnel barrier between the leads. Ni particles are embedded in the insulating layer. Figure 1b shows the top view of a typical device. The sandwich is located at the center of the figure where two Al leads overlap. A representative transmission electron microscope image of a Ni particle used in the device is shown in Fig. 1c. The particles were nucleated by depositing 0.5–0.6 nm of Ni on the Al_2_O_3_ surface. Using image analysis of 120 Ni neighboring particles we determined the particle base area distribution. Then, from the total deposited thickness, we obtain the volume distribution of the Ni particles 15 ± 6 nm^3^. The particles are single crystal and have an irregular surface, as can be seen in Fig. 1c. White lines indicate the primary crystal axes for face-centered-cubic Ni.

The fabrication process was described in our previous work^9^. The magnitude of the ground state spin (*S*) in the average particle volume can be estimated using the saturation magnetization at zero temperature in bulk Ni, 0.52 · 10^6^ A/m, leading to *S* = 420. Note that this estimate is subject to many uncertainties. The workfunction difference between Al and Ni, 1eV, will cause electron transfer from Al to Ni, until the electrochemical potentials of the particle and the leads are within ± half the charging energy (*E*_*C*_). In bulk Ni, the density of minority levels is much larger than the density of majority levels, so we expect that most of the transferred electron fill the minority levels, thereby reducing the spin roughly by 1*eEv*/*E*_*C*_ ∼ 100. This demonstrates fragile magnetism in Ni particles. Due to large surface-to-volume ratio and significant surface anisotropy, the validity of the macrospin model in the particle is also challenging^10^,^11^. Until now, similarly sized Co-particles in tunneling junctions have been qualitatively well understood in terms of the macrospin approach^7^,^8^,^12^,^13^,^14^,^15^,^16^, so here we will adopt that approach.

## Macrospin Model

Generally, in a quantum dot at temperatures much smaller than the charging energy, electron transport between the leads is sequential^17^. If the electron-box level spacing of the dot is large compared to the thermal energy, then the differential conductance versus bias voltage will exhibit peaks at voltages corresponding to the discrete energies of the tunneling transitions^18^,^19^. An addition of an electron into an electron-box level of a ferromagnetic particle, also results in an addition of a spin-orbit energy shift, which depends strongly on the direction of the magnetization and changes the total magnetic anisotropy of the particle^7^,^8^,^12^,^13^,^14^,^15^,^16^. In the macrospin model, the shift is a relatively simple and smooth function of the magnetization direction, without too many minima and maxima^15^,^16^. We consider an example where the macrospin Hamiltonians of the *N*− and *N* + 1-electron particle are 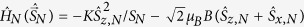 and 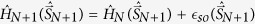, respectively. Here, *K* is the uniaxial anisotropy energy per spin, and 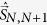 are the macrospin operators. *μ*_*B*_ is positive and equal to the Bohr magneton. The Zeeman energy has minimum when 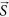 points along the magnetic field direction. The physical spin is 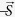, due to the negative sign of the electron g-factor. In the remainder of the text, when we use the word spin, we mean vector 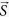. We assume that only one minority electron box level is involved, so that *S*_*N*+1_ = *S*_*N*_ − 1/2. The magnetic field (*B*) is in the x −  plane at the angle *π*/4 from the z-axis. The Stoner-Wohlfarth switching field for the *N*-electron particle (*B*_*sw*_) is *K*/2*μ*_*B*_. We assume that the spin-orbit shift is also uniaxial, 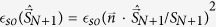, where 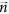 is the unity vector along the direction of the easy (hard) axis of the shift, and *ε*_*so*_ is the negative (positive) shift amplitude. In the example in this report, we use 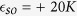, *S*_*N*_ = 150, and 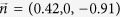. Figure 1d displays the classical energy surface for the *N*-electron particle as a function of the spin polar angles at *B* = 0.4*B*_*sw*_, showing a saddle point at 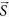 ≈ (148, 0, −26) and energy ≈−39 *K*.

**Figure 2:**
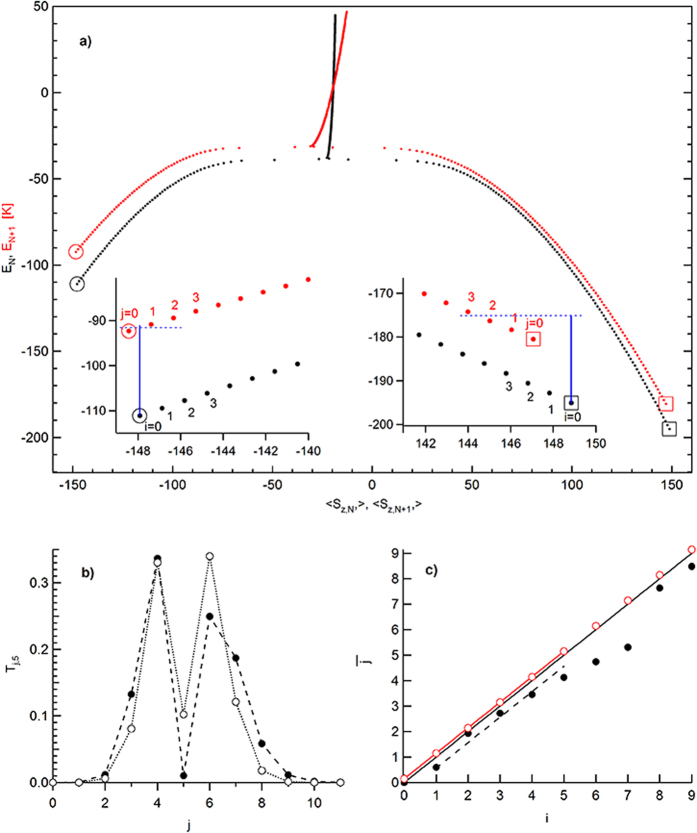
Eigenenergies and eigenstates of the model Hamiltonian: (**a)** Black and red dots are eigenenergies of the *N*− and the *N* + 1- electron particle, respectively. The bottom axis is the quantum average of the spin-z component in the eigenstates. Stable and metastable ground states are indicated by squares and circles, respectively. Left and right insets zoom in the vicinity of the metastable and the stable ground states, respectively. Vertical blue line represents the Fermi level. (**b)** Transition probability from initial eigenstate *i* = 5 to final eigenstates *j*, versus *j*. Filled (empty) circles correspond to the vicinity of the stable (metastable) ground state. (**c)** Average final state after a tunneling transition from initial state *i*, in the vicinity of the metastable ground state. Red and black circles are calculated at the Fermi level ∞ and that represented by the blue line in (**a)**, respectively. Full-red and dashed-black lines show the respective best linear fits. Full black line is defined as 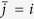. It delineates the regimes of magnetization diffusion at high Fermi level and magnetization relaxation at low Fermi level. In all panels, the magnetic field is set at 40% of the switching field.

Figure 2a displays the energy levels of the two Hamiltonians versus 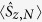 and 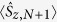, where 〈…〉 indicates the quantum-state average. The metastable and the stable ground states are indicated by the circles and the squares, respectively. The insets at bottom left and bottom right display the eigenenergies in the vicinity of the *N*− and the *N* + 1-electron metastable and stable ground states, respectively. The states are labeled using indices *i* and *j*, for the *N*− and the *N* + 1-electron particle, respectively. The ground states are *i, j* = 0, while the excited states *i, j* = 1, 2, 3, … are sorted with increasing energy. At energies above the saddle point, the eigenenergies increase sharply with 〈*S*_*z*,*N*_〉, as 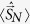 follows the ridge above the classical saddle point in Fig. 1d.

Next we consider electron tunneling. The tunneling probability between states *i* of the *N*-electron particle and states *j* of the *N* + 1-electron particle, is related to the square sum of the tunneling matrix elements,


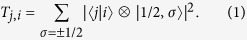


The calculation of *T*_*j*,*i*_ involves expressing the eigenstates in the basis |*S, S*_*z*_〉 and |*S* − 1/2, *S*_*z*_〉, followed by the Clebsch-Gordan decomposition of the product state |*i*〉 ⊗ |1/2, *σ*〉. For example, if the initial state is *i* = 5, above the metastable ground state, then Fig. 2b displays the transition probabilities *T*_*j*,5_. Only if *i* and *j* are very close, the transition probability will be significant. For large separations between states, we find *T*_*j*,*i*_ drops rapidly with | *j* − *i*|, typically as 10^−2|*j*−*i*|^.

The tunneling probability from the initial state *i* and the final state *j (P*_*i*,*j*_), per unit time, varies with the Fermi levels in the leads *E*_*F*,*l*_,


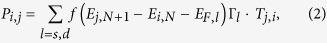


where Γ_*l*_ are the spin-independent tunneling rates to the source (*l* = *s*) and the drain (*l* = *d*) lead, and *f* is the Fermi function. Similarly, the tunneling probability from the initial state *j* and the final state *i* is


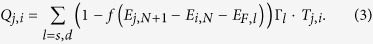


In the simulations, we generate random sequences of tunneling transitions *i* → *j* → *i*′ → *j*′… according to the above probabilities. Except for the tunnel coupling to the leads, we assume that no other damping between the macrospin and the environment. We further assume Γ_*d*_ = 100Γ_*s*_ and zero occupancy in the drain *f*(*E*_*j*,*N*+1_ − *E*_*i*,*N*_ − *E*_*F*,*d*_) = 0, so that the electron flow is mostly one-directional, from the source to the particle to the drain. The integration time is 5 · 10^5^/Γ_*s*_, which is comparable to the experimental timescale per data point. The thermal energy is assumed to be *k*_*B*_*T* = 0.1 *K*. From now on, the charging, the orbital, and the exchange energy of the tunneling transitions will be subtracted from the Fermi levels, for notational clarity. In our model, such energy contributions are assumed not to play a role on spin dynamics.

Assume first that the Fermi level in the source lead (*E*_*F*_) is so large that the Fermi function is equal to 1. Consider the vicinity of the metastable ground state of the *N*-electron particle, defined by the range *i* < 5. Upon a tunneling transition from state *i* to the various final states *j* of the *N* + 1-electron particle, the average final state can be calculated as 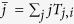 and is displayed in Fig. 2c by the open red circles. 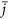 is linear with *i*, with the best fit 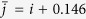 indicated by the red line. Similarly, if *i* is in such vicinity about the stable ground state, we obtain linear dependence for the average excited state, 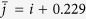. The removal of an electron from the particle can also excite the particle magnetically. For the initial states *j* in such vicinities of the ground states of the *N* + 1-electron particle (*j* < 5), we obtain that the average final state of the *N*-electron particle is linear with the initial state, that is, 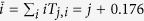 (*j* + 0.252), about the metastable (stable) ground state. Overall, in sequential electron tunneling at large *E*_*F*_, *i* increases on average by 0.322 (0.481) about the metastable (stable) state, per tunneling cycle. As a result, the magnetization exhibits diffusion, reaching equipartition among the states with a given *N*. Such an effect was first studied by Waintal and Brouwer in the context of tunable magnetic relaxation time^20^, in the regime without spin-orbit scattering.

The necessary condition for magnetic inversion is that the energy differences *E*_*j*,*N*+1_ − *E*_*i*,*N*_ be higher above the metastable ground state than about the stable one. This condition is satisfied here as can be seen in Fig. 2a. Statistically, averaged over many electron tunneling sequences, the energy will not be deposited into the magnetic system, if *E*_*F*_ < 20.7 *K* and the magnetization is initially in the metastable ground state. As an example, consider the Fermi level of *E*_*F*_ = 20 *K*, which corresponds to the lengths of the full blue lines in the insets of Fig. 2a. As indicated by the dashed blue line in the right inset, if the particle is initially in the stable ground state, *i* = 0, then there will be sufficient *E*_*F*_ to tunnel in to the 2nd excited state of the *N* + 1-electron particle, but not into the third excited state. Despite the reduced *E*_*F*_, we calculate that the energy will be injected into the magnetic system leading to magnetization diffusion away from the stable ground state, but with somewhat reduced rate compared to that at large *E*_*F*_. On the other hand, if the particle is initially in the metastable ground state *i* = 0, *E*_*F*_ is below the first excited state of the *N* + 1-electron particle, as shown in the left inset in Fig. 2a. After tunneling on, the magnetization remains in the metastable ground state.

Consider the vicinity *i* < 5 about the metastable ground state of the *N*-electron particle at *E*_*F*_ = 20 *K*. Figure 2c shows that, upon an electron tunneling into the particle, the average excited state 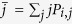 is smaller than *i*, in contrast to the regime at high *E*_*F*_. Now, the tunneling transitions relax the magnetization to the metastable ground state. The best linear fit, indicated by the dashed black line in Fig. 2c, is 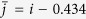. At the same *E*_*F*_, if the magnetization were initially about the stable ground state, the energy transfer into the magnetic system would remain positive, specifically, 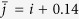. Overall, in sequential electron tunneling at *E*_*F*_ = 20 *K, i* increases on average by −0.258 (+0.392) per tunneling cycle, above the metastable (stable) state. The magnetization rapidly diffuses away from the stable ground state and relaxes towards the metastable ground state. With further reduction in *E*_*F*_, the current drops to zero as the magnetization is fully relaxed, reaching inversion.

**Figure 3:**
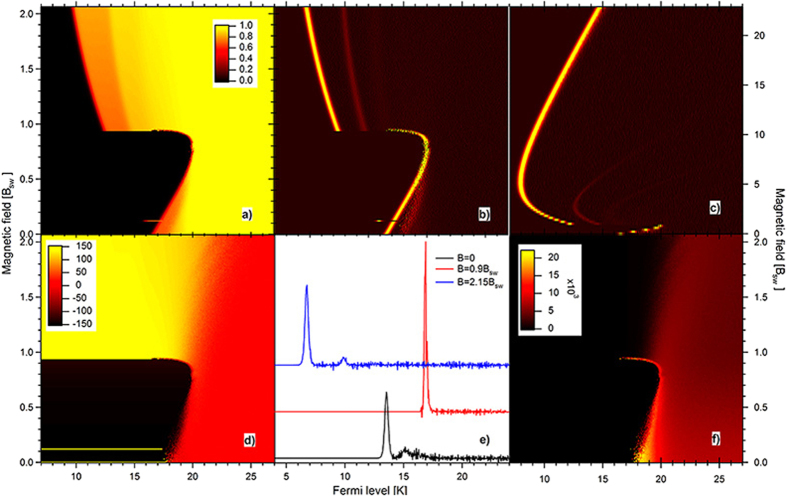
Numerical simulations: (**a**,**b)** Current (in units of *e*Γ_*s*_) and differential conductance maps with Fermi level and magnetic field, respectively. The magnetic field is in units of the Stoner-Wohlfarth switching field of the *N*-electron particle. The Fermi level is in units of the anisotropy energy per spin. (**c)** The conductance map in wider magnetic field range, showing crossover to the linear regime in strong magnetic field. (**d)** Map of the particle time averaged spin-z component, showing magnetic inversion at low Fermi level and below the switching field. (**e)** Conductance traces at three different fields obtained from (**b)**, offset for clarity. (**f)** Map of the variance of the spin z-component, showing enhancement of magnetic noise in the regime of inverted relaxation.

Figure 3 displays the results of the simulations. Figure 3a,b present the tunneling current and the differential differential conductance map versus source Fermi level and magnetic field. The Fermi level is varied from high to low while the magnetic field is fixed. The conductance map in Fig. 3b displays peaks as a function of *E*_*F*_. Figure 3e displays three conductance traces at fixed magnetic field, taken from the conductance map in Fig. 3b. The noise at high *E*_*F*_ is due to current shot-noise. At zero magnetic field, the conductance peaks exhibit splitting, or ZFS, with energy close to the FMR energy of 2 *K*. ZFS decreases with magnetic field, with a near degeneracy in the magnetic field range (0.6, 1)*B*_*sw*_, as exemplified by the trace at *B* = 0.9*B*_*sw*_ in Fig. 3e. In the magnetic field above the switching field, the splitting is resolved in the form of nonlinear Zeeman splitting.

In the low magnetic field range, Fig. 3b shows that the peak Fermi levels vary rapidly and nonmonotonically with the magnetic field. There is a discontinuity in the peak Fermi levels lightly before the Stoner-Wohlfarth switching field. The nonlinear Zeeman splitting above that field extrapolates to the FMR energy at zero magnetic field, as could be inferred by closely looking at Fig. 3b. In the strong magnetic field regime, Fig. 3c shows a crossover to the linear dependence of peak *E*_*F*_ versus magnetic field. The amplitude of the weaker Zeeman partner diminishes rapidly with the magnetic field. The crossover field between the linear and the nonlinear regimes is given by 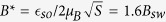 in this simulation.

Figure 3d displays the time-average spin-z component of the particle versus Fermi level and magnetic field. Below the magnetic switching field, the magnetization populates the quantum states around the metastable minimum, as the Fermi level is reducing across the tunneling transition energy range. Such inversion is indicated by the large negative value of 〈*S*_*z*_〉 at low *E*_*F*_. Metastability of the magnetization results in the negative magnetic field dependence of the ZFS below *B*_*sw*_ in Fig. 3b. The inverting is also manifested by an increased magnetic noise, which is displayed in Fig. 3f. In the magnetic field above the switching field, the magnetic inversion does not take place. We have studied a wide range of macrospin Hamiltonians and obtained parameter maps of the inversions. The maps are provided in supplementary material S1. In the Hamiltonians that do not exhibit magnetic inversion, ZFS increases with the magnetic field as the magnetization is localizing about the stable ground state. Thus, the magnetic field dependence of the ZFS could be used to identify the inversion.

## Experiment

**Figure 4:**
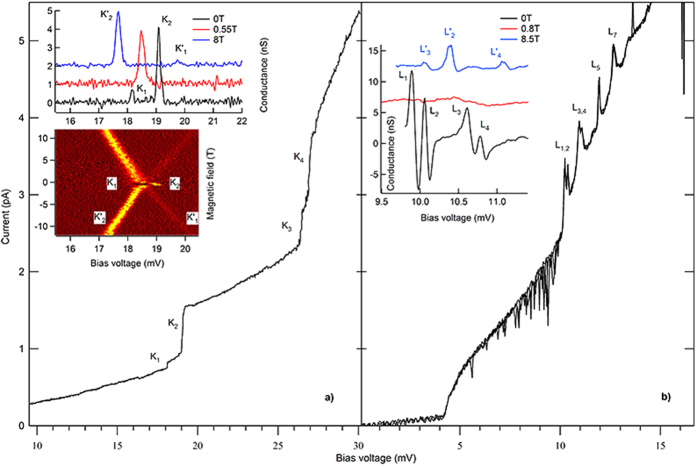
Current-Voltage measurements, discrete levels at *T* = 30 mK, and zero-field splitting: (**a)** Sample 1. Steps *K*_1−4_ indicate discrete levels. Upper graph in the inset displays differential conductance versus bias voltage in the vicinity of levels *K*_1_ and *K*_2_, at three magnetic fields. The traces are offset by 1nS for clarity. Lower image in the inset is the conductance map with voltage and magnetic field, showing that *K*_1_ and *K*_2_ resolve into Zeeman splitting between 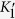 and 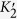 in the strong magnetic field. (**b)** Sample 2. Peaks *L*_1−7_ indicate discrete levels followed by regions of negative differential conductance. The inset displays differential conductance traces in the vicinity of levels *L*_1_ − *L*_4_, at three magnetic fields. The traces are offset by 6nS for clarity. Zero field splitting between levels *L*_3_ and *L*_4_ resolves into nonlinear Zeeman splitting between levels 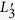 and 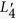. In both (**a**,**b)**, the splitting is significantly reduced and cannot be resolved in nonzero magnetic field, indicating near degeneracy, as shown by the red lines in the insets.

In this report we present results on two samples that display negative magnetic field dependence of the ZFS, which is considetnt with magnetic inversions. Current vs. voltage (IV) measurement in sample 1 is shown in Fig. 4a. The mixing-chamber temperature during the measurement is 30 mK. Discrete steps in the IV labeled *K*_1_-*K*_4_ are indicated. The splitting between *K*_1_ and *K*_2_ in voltage, 0.956 mV, is converted to energy Δ_1_ = 0.407 meV, after correcting for capacitive division, which is determined from the ratio od the Coulomb-blockade voltages at positive and negative bias. This splitting value is within the range of the ZFS measured in single magnetic molecules^5^,^6^. The splitting between levels *K*_3_ and *K*_4_ is 0.247 meV. The upper graph in the inset in Fig. 4a displays differential conductance versus bias voltage, in the vicinity of levels *K*_1_ and *K*_2_. The splitting is reduced when the magnetic field is increased, and cannot be resolved experimentally in the magnetic field interval (0.5, 1.5)T, as indicated by the trace at 0.55T in the inset in Fig. 4a. But above that magnetic field interval, the splitting resolves in the form of Zeeman splitting between levels 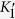 and 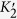, as shown by the conductance map in the lower panel of the inset in Fig. 4a, with the g-factor of 1.98. Thus, ZFS between levels *K*_1_ and *K*_2_ can be viewed as the continuation of the Zeeman splitting in the strong magnetic field, demonstrating the magnetic origin of the ZFS.

Figure 4b displays three superimposed IV measurements in sample 2. There is a bias voltage region (4, 10) mV with significant noise. The effects described herein will be presented outside the noisy voltage region. The origin of the noise is likely due to charge noise and unrelated to the magnetic properties of the particle. The IV exhibits levels indicated by *L*_1−7_, which are followed by the regions of negative differential conductance. Such regions are found in the low magnetic field range, *B* < 0.3T, and may be do to the superconducting density of states in the Al leads^18^. But negative differential conductance is also found in the high magnetic field range, >1.5T, as will be shown further below. Tunneling studies of Mn12 magnetic molecules find similar negative differential conductance, due to a current blocking effect involving nondegenerate spin multiplets^21^. The splittings between *L*_1_ and *L*_2_ and *L*_3_ and *L*_4_, are 0.148 mV, and the corresponding ZFS energy is Δ_2_ = 0.091 meV. In the magnetic field range (0.3, 1.5)T the levels are too broad to observe any splitting, as can be seen from the conductance trace at 0.8T in Fig. 4b. In the strong magnetic field, the splittings of the conductance peaks reemerge in the form of nonlinear Zeeman splitting, to be described in the second paragraph below. The conductance trace measured at 8.5T displayed in Fig. 4b, displays a Zeeman split pair 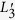 and 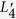, which is connected to the ZFS pair *L*_3_ and *L*_4_ at *B* = 0T. Level 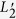 is connected to the pair of levels *L*_1_ and *L*_2_ at *B* = 0, however the location of the lower energy Zeeman partner of 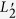 is expected to be in the noisy voltage region in the strong magnetic field, and cannot be identified.

**Figure 5:**
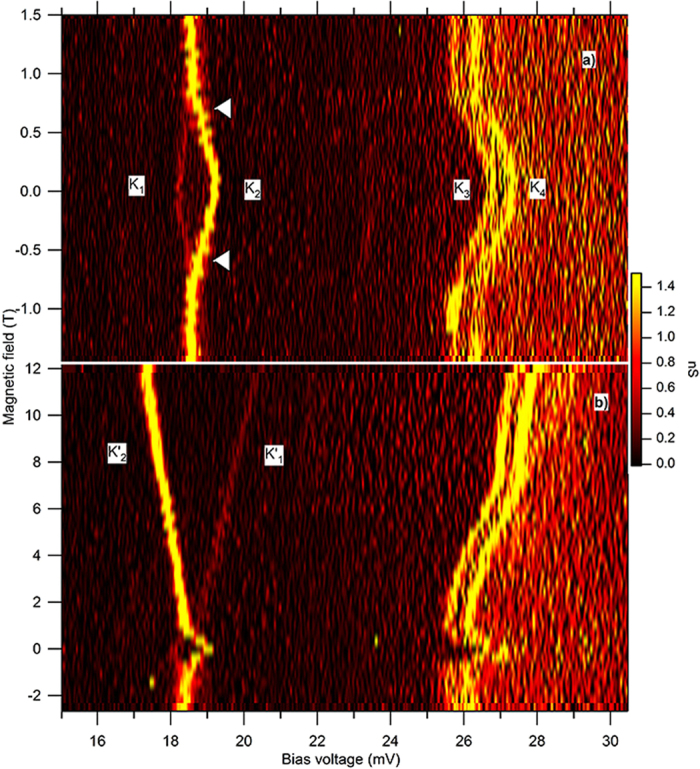
Conductance maps versus voltage and magnetic field in sample 1. (**a)** Low magnetic field region displays zero field splitting between levels *K*_1_ and *K*_2_, which decreases with magnetic field, approaching degeneracy at magnetic field of approximately 0.5T or slightly above (white arrows). (**b)** Continuity between zero field splitting and Zeeman splitting, between 17 and 20 mV.

Figure 5a shows a detailed conductance map with bias voltage and magnetic field in the low magnetic field region in sample 1. To obtain such maps, the bias voltage is ramped fast in a triangle wave, while the magnetic field is swept slowly. The tunneling spectra display no magnetic hysteresis. The splitting between levels *K*_1_ and *K*_2_ is parabolic about *B* = 0. Near degeneracy is approached at ≈±0.5T as shown by the white triangular arrows. Above that magnetic field, the levels remain close in energy. Within our energy resolution it is not possible to determine if levels *K*_1_ and *K*_2_ cross or avoid crossings. The crossings are not simply linear, because above 0.5T the levels separate much more slowly with magnetic field than below that field. This property is consistent with the simulation in Fig. 3b,e, which also show a magnetic field region of near degeneracy in the magnetic field range (0.6, 1)*B*_*sw*_. Figure 5b shows that levels *K*_3_ and *K*_4_ have magnetic field independent splitting, which indicates that their splitting has nonmagnetic origin. They exhibit a parabolic-like field dependence at low magnetic field, and a much weaker magnetic field dependence at and above 0.5T. The differential conductance in sample 1 is positive.

**Figure 6:**
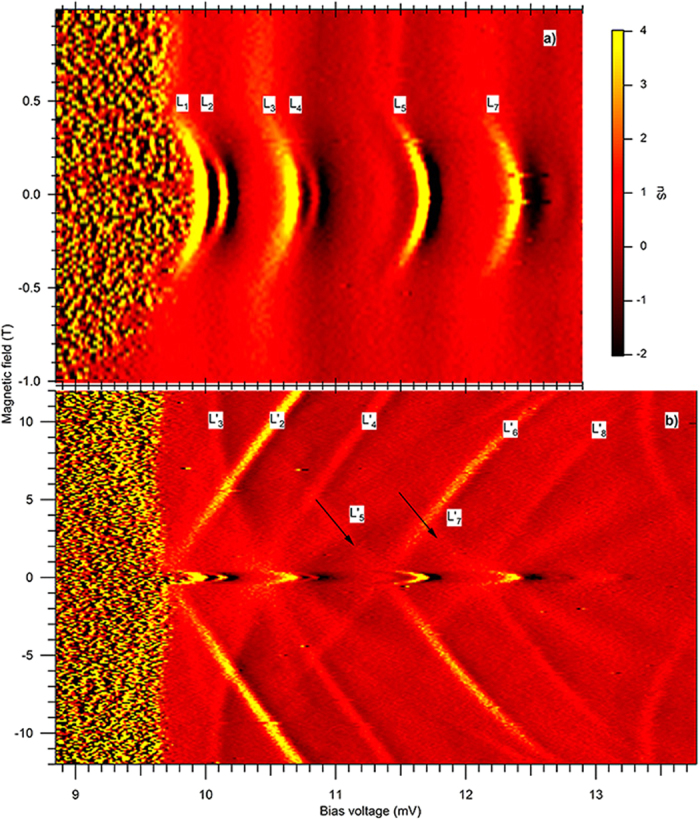
Conductance maps versus voltage and magnetic field in sample 2. (**a)** Low magnetic field region displays zero field splitting of Levels *L*_1_ and *L*_2_ and *L*_3_ and *L*_4_, which decrease by 30% when the magnetic field is increased from zero to 0.25T. (**b)** Continuity between zero field splitting and Zeeman splitting in wider magnetic field range. Nonlinear Zeeman splitting between levels 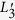 and 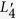 in the strong magnetic field is a continuation of the zero field splitting between levels *L*_3_ and *L*_4_.

Figure 6 displays the conductance maps in sample 2. The regions of negative differential conductance, which are located at voltages immediately after the conductance peaks, are present in both low and strong magnetic fields. In Fig. 6a, the splitting between *L*_1_ and *L*_2_ is parabolic about *B* = 0 and is reduced from 0.091 meV to 0.62 meV as the magnetic field increases from zero to 0.23T. The degree of degeneracy cannot be demonstrated in this sample due to the significant peak broadening above that field. Figure 6b shows nonlinear Zeeman splitting between levels 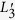 and 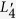, which are continuously connected to levels *L*_3_ and *L*_4_ at zero magnetic field. Levels 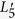 and 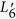 are also a Zeeman split pair, and they are a continuation of level *L*_5_ at zero magnetic field.

## Discussion and Conclusions

In two Ni particles we have measured ZFS due to magnetic excitations, with the property that the excitation energy decreases rapidly with the magnetic field, and in one sample approaches near degeneracy at nonzero magnetic field, within our energy resolution. To the extent that the magnetic excitation can be identified as the elementary excitation of the Ni particle macrospin, these findings demonstrate that the magnetization is localizing in the vicinity of the metastable anisotropy energy minimum, during the measurement of the ZFS. Using the macrospin model, we have shown that such inversion of magnetization is theoretically possible, and it reproduces qualitatively our key experimental signatures displayed in Figs 4, 5 and 6.

There are several data features that are dissimilar with our macrospin simulations. Our measurements do not display any discontinuity in energy levels, while the macrospin simulations display discontinuity at the switching field. Instead, in our measurement the transition from ZFS to near degeneracy at nonzero magnetic field, and from the near degeneracy to Zeeman spitting in the strong magnetic field, appears to be continuous. In addition, the weight of the weaker partner in sample 1 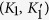 decreases gradually with magnetic field. In the magnetic field interval ±0.2T, the weight of the weak partner, *K*_1_, is 12% relative to the weight of the strong partner, *K*_2_. This weight decreases to approximately 9% and 6%, for partner 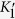 at 3T and 9T, respectively. In comparison, the macrospin model predicts that the weight of ZFS levels redistribute continuously below the switching field, followed by the discontinuity in weights at the switching field. In the linear regime in the strong magnetic field, the model predicts much stronger suppression of the weaker Zeeman partner compared to our measurement, as can be seen for example by comparing Figs 3c and 5b. The implication is that the matrix elements of the tunneling Hamiltonian, or *T*_*j*,*i*_, decrease much more slowly with the magnetic field than what the macrospin model predicts for the linear field regime. Further work will explore the role of noncollinearity of spins due to strong surface anisotropy^10^,^11^, and their effects on various tunneling transition probabilities and spin dynamics, to improve the similarity between theory and experiment.

Inverted magnetization suggests a new kind of magnetic switching device analogous to spin-transfer-torque switches^22^,^23^,^24^,^25^, but would not require any spin-polarized carriers in the leads. The new device would require spin-orbit engineering in the particle or a magnetic molecule, to enable control of the magnetization relaxation rates. Such a device would require the ability to create both inverted and equilibrium magnetization, which could be achieved for example by changing the sign of the bias voltage or by applying a gate voltage, in order to provide sequential tunneling regime where the lowest electron-box level favors particular magnetization. Then, the magnetization could be written in the metastable or the stable state by applying a voltage pulse, depending on which electron-box level were selected.

## Additional Information

**How to cite this article**: Jiang, W. *et al*. Evidence of Magnetic Inversion in Single Ni Nanoparticles. *Sci. Rep.* **6**, 36156; doi: 10.1038/srep36156 (2016).

**Publisher’s note:** Springer Nature remains neutral with regard to jurisdictional claims in published maps and institutional affiliations.

## Supplementary Material

Supplementary Information

## References

[b1] Néel L. Théorie du traînage magnétique des ferromagnétiques en grains fins avec applications aux terres cuites. Ann. Géophys 5, 99 (1949).

[b2] Brown W. F. Thermal fluctuations of a single-domain particle. Phys. Rev. 130, 1677–1686 (1963).

[b3] Bonet E. . Three-dimensional magnetization reversal measurements in nanoparticles. Phys. Rev. Lett. 83, 4188 (1999).

[b4] Wernsdorfer W. Classical and quantum magnetization reversal studied in nanometer-sized particles and clusters. Adv. Chem. Phys. 118, 99–190 (2001).

[b5] Jo M.-H. . Signatures of molecular magnetism in single-molecule transport spectroscopy. Nano Letters 6, 2014–2020 (2006). 16968018 10.1021/nl061212i

[b6] Zyazin A. S. . Electric field controlled magnetic anisotropy in a single molecule. Nano. Lett. 9, 3307 (2010). 10.1021/nl1009603 20687519

[b7] Deshmukh M. M. . Magnetic anisotropy variations and nonequilibrium tunneling in a cobalt nanoparticle. Phys. Rev. Lett 87, 226801 (2001). 11736415 10.1103/PhysRevLett.87.226801

[b8] Gueron S., Deshmukh M. M., Myers E. B. & Ralph D. C. Tunneling via individual electronic states in ferromagnetic nanoparticles. Phys. Rev. Lett. 83, 4148 (1999).

[b9] Wei Y. G., Malec C. E. & Davidovic D. Saturation of spin-polarized current in nanometer scale aluminum grains. Phys. Rev. B 76, 195327 (2007).

[b10] Garanin D. A. & Kachkachi H. Surface contribution to the anisotropy of magnetic nanoparticles. Phys. Rev. Lett 90, 065504 (2003). 12633301 10.1103/PhysRevLett.90.065504

[b11] Kachkachi H. & Bonet E. Surface induce cubic anisotropy in nanomagnets. Phys. Rev. B 73, 224402 (2006).

[b12] Jiang W., Birk F. & Davidovic D. Effects of confinement and electron transport on magnetic switching in single co nanoparticles. Scientific Reports 3, 1200 (2013). 23383370 10.1038/srep01200 PMC3563040

[b13] Gartland P., Jiang W. & Davidovic D. Voltage control of magnetic hysteresis in a nickel nanoparticle. Phys. Rev. B 91, 235408 (2015).

[b14] Usaj G. & Baranger H. U. Anisotropy in ferromagnetic nanoparticles: Level-to-level fluctuations of a collective effect. Europhys. Lett. 72, 110 (2005).

[b15] Cehovin A., Canali C. M. & MacDonald A. H. Magnetization orientation dependence of the quasiparticle spectrum and hysteresis in ferromagnetic metal nanoparticles. Phys. Rev. B 66, 094430 (2002).

[b16] Brouwer P. W. & Gorokhov D. A. Bound on anisotropy in itinerant ferromagnets from random impurities. Phys. Rev. Lett. 95, 017202 (2005). 16090649 10.1103/PhysRevLett.95.017202

[b17] Averin D. V. & Likharev K. K. In Altshuler B. L., Lee P. L. & Webb R. A. (eds) Mesoscopic Phenomena in Solids 169 (Elsevier and Amsterdam, 1991).

[b18] Ralph D. C., Black C. T. & Tinkham M. Spectroscopic measurements of discrete electronic states in single metal particles. Phys. Rev. Lett. 74, 3241 (1995). 10058147 10.1103/PhysRevLett.74.3241

[b19] Davidović D. & Tinkham M. Spectroscopy, interactions, and level splittings in an nanoparticles. Phys. Rev. Lett. 83, 1644–1647 (1999).

[b20] Waintal X. & Brouwer P. W. Tunable magnetic relaxation mechanism in magnetic nanoparticles. Phys. Rev. Lett. 91, 247201 (2003). 14683151 10.1103/PhysRevLett.91.247201

[b21] Heersche H. B. . Electron transport through single *mn*12 molecular magnets. Phys. Rev. Lett. 96, 206801 (2006). 16803192 10.1103/PhysRevLett.96.206801

[b22] Slonczewski J. C. Current-driven excitation of magnetic multilayers. Journal of Magnetism and Manetic Materials 159, L1–L7 (1996).

[b23] Berger L. Emission of spin waves by a magnetic multilayer traversed by a current. Phys. Rev. B 54, 9353–9358 (1996). 10.1103/physrevb.54.9353 9984672

[b24] Sun J. Current-driven magnetic switching in manganite trilayer junctions. Journal of Magnetism and Magnetic Materials 202, 157 (1999).

[b25] Katine J. A. . Current-driven magnetization reversal and spin-wave excitations in co/cu/co pillars. Phys. Rev. Lett. 84, 3149 (2000). 11019034 10.1103/PhysRevLett.84.3149

