# Supplementary material for: Evidence of Magnetic Inversion in Single Ni Nanoparticles
Source: Sci Rep. 2016 Nov 8;6:36156. doi: 10.1038/srep36156 (PMC5099931; doi:10.1038/srep36156)
Supplement: Supplementary Information [file srep36156-s1.pdf]

Supplementary material S1

Evidence of Magnetic Inversion in Single Ni Nanoparticles.

Authors: W. Jiang, P. Gartland, and D. Davidović

School of Physics, Georgia Institute of Technology, Atlanta, GA 30332, U.S.A.

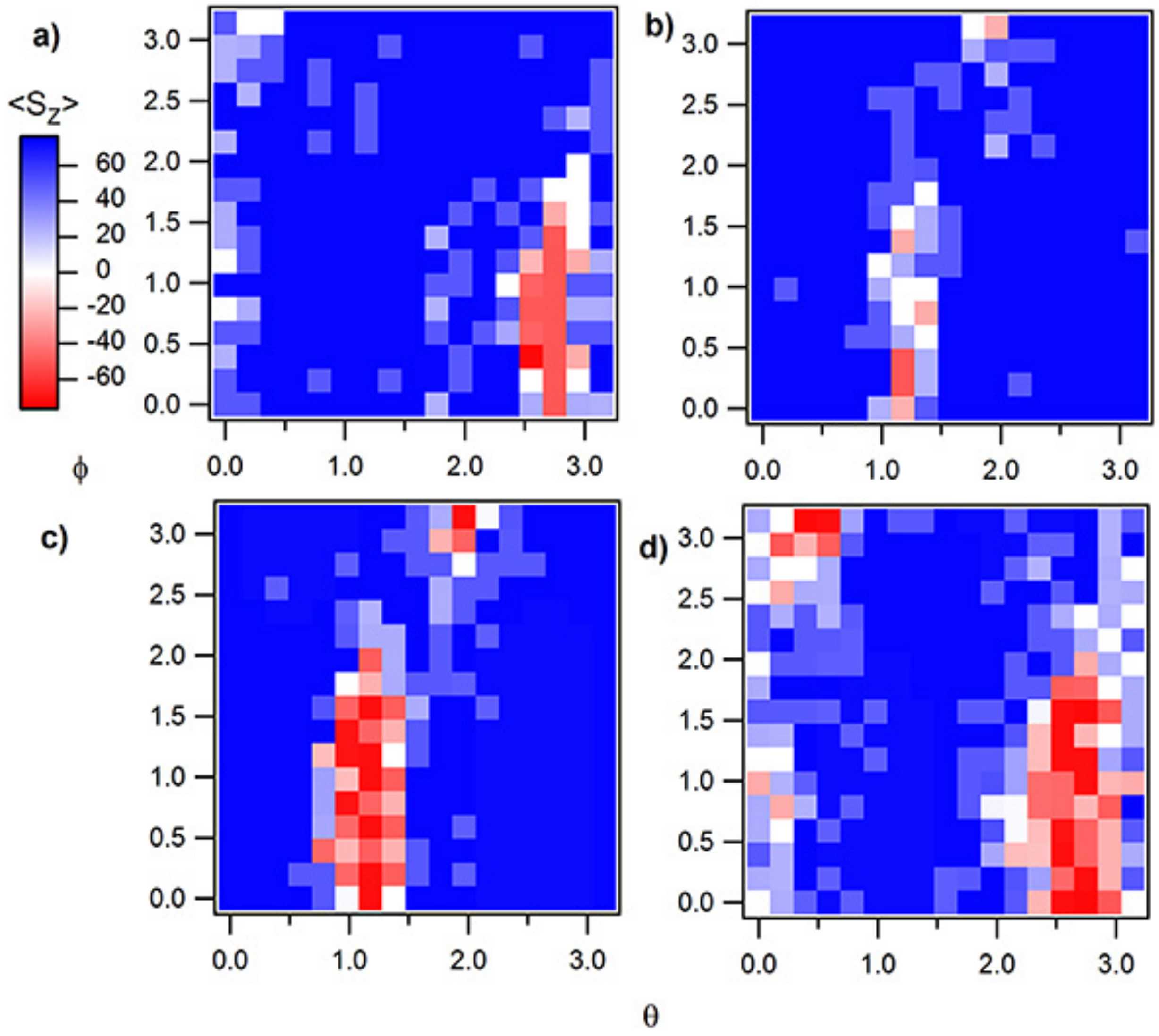

FIG. 1. Anisotropy maps of magnetic inversions. The magnetic field is applied along direction specified by the conventional polar angles, $\phi = \pi/4$ and $\theta = \pi/4$. The magnetic Hamiltonians and the tunneling rates are given in the main text. The polar angles in **a-d** correspond to the unit vector $\vec{n}$ of the uniaxial single-electron anisotropy (invariant with respect to inversion, $\phi \to \phi + \pi$ and $\theta \to \pi - \theta$). Here, the ground state spin of the N-electron particle is $S_N = 75$. The average spin z-component is calculated after the Fermi level is slowly reduced from high to low value, relative to the tunneling transition energy. That is, $\langle S_z \rangle$ is the frozen spin-z component after the current is reduced to zero. The magnetic field is varied in the range $0.1 - 0.9 B_{sw}$ and $\langle S_z \rangle$ is averaged over that range. The inversions are indicated by the red regions. Nearly identical maps were found for the case of biaxial anisotropy of the N-electron particles. **a** and **b**: $\epsilon_{so} = 20K$ and $-20K$, respectively. E_F is varied in the source lead while $E_F = -\infty$ in the drain. Electron initially tunnels into the particle from the source. **c** and **d**: $\epsilon_{so} = 20K$ and $-20K$, respectively. E_F is varied in the drain while $E_F = +\infty$ in the source. Electron initially tunnels into the drain from the particle.
